# Predictive value of N-terminal pro-B-type natriuretic peptide (NT-pro BNP) combined with D-dimer for no-reflow phenomenon in patients with acute coronary syndrome after emergency of percutaneous coronary intervention

**DOI:** 10.1080/21655979.2021.1988361

**Published:** 2021-10-26

**Authors:** Yujing Diao, Meifeng Yin, Baoguo Zhang, Bin Sun

**Affiliations:** a Department of Emergency, Yidu Central Hospital of Weifang, Weifang, Shandong, China; b Clinical Internal Medicine, Qingzhou Mihe Central Health Center, Shandong, Weifang, China

**Keywords:** Acute coronary syndrome, percutaneous coronary intervention, NT-proBNP, D-dimer, no-reflow phenomenon

## Abstract

Acute coronary syndrome (ACS) is a critical illness in cardiovascular disease. The purpose of this study was to investigate the value of N-terminal pro-B-type natriuretic peptide (NT-proBNP) and D-dimer in predicting the occurrence of no reflow in emergency percutaneous coronary intervention (PCI) in patients with ACS. One hundred and sixty-eight ACS patients were recruited, including 88 patients with normal reflow and 80 patients with no reflow after emergency PCI. The levels of serum NT-proBNP and D-dimer in the patients were detected before PCI, immediately after PCI, 2 hours, and 6 months after PCI. The ROC curve was used to evaluate the predictive value of NT-proBNP and D-dimer in no-reflow phenomenon. Logistic regression model was used to analyze the independent influencing factors of no reflow phenomenon. Logistic regression analysis confirmed that NT-proBNP and D-dimer were independent predictors of the occurrence of no reflow in the total population. The ROC curve showed that the AUC value was 0.909 when NT-proBNP combined with D-dimer. The detection of NT-proBNP combined with D-dimer was helpful to predict the occurrence of no-reflow phenomenon after emergency PCI in ACS patients.

## Introduction

Acute coronary syndrome (ACS) is a critical illness in cardiovascular disease. ACS is a group of clinical syndromes caused by acute myocardial ischemia, including unstable angina, non-ST-segment elevation myocardial infarction (NSTEMI), and ST-segment elevation myocardial infarction (STEMI) [1,2]. Among them, unstable atherosclerotic plaque rupture leads to partial or complete occlusion of the epicardial coronary artery, which is the most important pathogenesis of ACS [3]. Several ACS patients experience symptoms such as fatigue, chest discomfort, shortness of breath, and irritability during activities. In severe cases, syncope or sudden death may occur, and timely medical treatment is required [1,4].

After more than 30 years of rapid development, percutaneous coronary intervention (PCI) has become the most common technique for the treatment of coronary artery disease since its inception in 1977 [5]. Compared with thrombolytic therapy, PCI reperfusion therapy for patients with acute myocardial infarction has been shown to be effective in improving clinical outcomes [6]. It has been reported that 30%~40% of patients undergoing primary PCI may have no reflow, although there is no obvious spasm, thrombus, or severe residual stenosis [7]. At present, it is generally believed that the pathological mechanism leading to the no-reflow of patients after surgery is distal embolization, ischemic injury, and susceptibility of coronary microcirculation to injury [8]. No-reflow phenomenon has a very important clinical significance, which is independently related to patient mortality and the incidence of malignant arrhythmias [9]. Although potential predictors of no-reflow phenomenon have been reported previously, such as platelet/lymphocyte ratio [10], more predictors are urgently needed in clinical practice to overcome this problem. N terminal pro-B-type natriuretic peptide (NT-proBNP) is a neurohormone synthesized and released by ventricular muscles, which is increased in acute myocardial infarction and angina pectoris [11]. Hong et al. reported that the serum level of NT-proBNP in patients with no reflow was significantly higher than that in patients with normal reflow, suggesting that preoperative NT-proBNP may be a powerful predictor for the no-reflow phenomenon after PCI in patients with acute myocardial infarction [4,12]. D-dimer is an indicator reflecting the degree of coagulation, fibrinolytic activation, and thrombosis in coronary artery disease [13]. In AMI patients, high levels of D-dimer are often associated with increased cardiovascular mortality after PCI [14]. Ayhan et al. have shown a correlation between D-dimer levels and no-reflow phenomena [15].

Previous studies have shown that NT pro-BNT and D-Dimer are, respectively, associated with no-reflow phenomena to a certain extent. And NT pro-BNT has potential predictive value for no reflow after PCI. However, the predictive value of a single biomarker for disease is often limited. Therefore, in this study, to explore the predictive value of the no-reflow phenomenon in ACS patients after PCI, we combined NT-proBNP and D-dimer for the first time. Our study showed that the predictive value of the combination of NT pro-BNT and D-Dimer for no reflow is correspondingly higher than NT pro-BNT or D-Dimer alone.

## Materials and methods

### Study population

This study has been ethically reviewed and approved by the ethics Committee of Yidu Central Hospital in Weifang. All recruited individuals and their family members have given informed consent and voluntarily participated in the study.

The subjects recruited in this study were all patients with ACS who came to Yidu Central Hospital of Weifang for emergency PCI. Inclusion criteria: 1) the diagnosis of ACS is based on specific electrocardiograms (ECG) standards established by the European Society of Cardiology, American College of Cardiology, American Heart Association, and World Heart Federation (ESC/ACCF/AHA/WHF) committees [16]; 2) the criteria for determining no-reflow phenomenon after PCI is based on the thrombolysis in myocardial infarction (TIMI) flow grade, which requires TIMI flow grade <3 and no obvious stenosis or vasospasm. According to this standard, patients were classified as no-reflow patients and normal reflow patients. Exclusion criteria: 1) patients with coronary artery spasm thromboembolism after coronary artery bypass surgery; 2) patients who had undergone major surgery or had a history of ischemic stroke in the last 3 months; 3) patients with failed thrombolysis; 4) patients with other serious diseases, such as cardiomyopathy, severe anemia, autoimmune disease, severe hepatorenal insufficiency, and so on; 5) patients with malignant tumors; 6) patients with active infection; 7) patients who did not want to participate in the study. After the exclusion of unqualified subjects according to the above criteria, a total of 168 eligible subjects participated in this study, including 80 patients with no-reflow and 88 patients with normal reflow. Moreover, all subjects enrolled in the study underwent a physical examination to record their age, gender composition, body mass index (BMI), hypertension, and other medical conditions.

### Sample collection and detection

Venous blood from all ACS patients was collected in a centrifuge tube before emergency PCI, immediately after PCI, 2 hours after PCI, and 6 months after PCI. After rapid centrifugation, the serum was collected and stored in a refrigerator at −80°C for later use. Serum NT-proBNP was detected by immunofluorescence double antibody sandwich method with an Elecsys 2010 analyzer (Roche Diagnostics, Mannheim, Germany) according to a previously published method [17]. D-dimer was quantitatively determined by an enzyme-linked immunosorbent assay (ELISA) with a microplate reader (SpectraMax; Molecular Devices, USA). The above experiments were carried out in accordance with the operating instructions of relevant kits and instruments.

### Statistical analysis

All statistical analyses in this paper were performed using SPSS 20.0 software (SPSS Inc., Chicago, IL) and GraphPad Prism 6.0 software (GraphPad Software, Inc., USA). Independent sample *t* test was used for intergroup comparison, and one-way ANOVA was used for multi-group comparison. Logistic regression analysis was used to analyze the risk factors of no-reflow after PCI in ACS patients. Receiver operating characteristic (ROC) curve was used to evaluate the diagnostic value of NT-proBNP and D-dimer in no-reflow patients. Data were presented as mean ± SD, and *P* < 0.05 was considered statistically significant.

## Results

### Clinical characteristics of study population

A total of 168 ACS patients were recruited in this study, which were divided into two groups according to whether no-reflow occurred after PCI, including normal reflow patients (n = 88) and no-reflow patients (n = 80). The clinical characteristics of the study population are shown in Table 1. There were no statistically significant differences in gender composition, age, BMI, hypertension, diabetes, hyperlipidemia, smokers, and other indicators (*P* > 0.05). However, it was worth mentioning that the average levels of NT-proBNP and D-dimer in the no-reflow patient group were higher than those in the normal reflow patient group (*P* < 0.05), which preliminarily suggested that these two factors may be risk factors in no-reflow patients. Besides, there was a statistically significant difference in Killip class ≥ II, SYNTAX score, and CRP (*P* < 0.001).

**Table 1.** Clinical data of the study population

Variables	All subjects (N = 168)		*P* value
	Reflow group (n = 88)	No reflow group (n = 80)	
N (Male and female)	40/48	39/41	0.669
Age (years)	57.84 ± 3.85	57.59 ± 4.36	0.648
BMI (kg/m^2^)	21.49 ± 0.89	21.50 ± 0.87	0.289
Smoker (n)	51/37	35/45	0.066
Killip class ≥ II on admission	17/71	25/55	<0.001
Baseline SYNTAX score	14.76 ± 6.97	18.59 ± 7.21	<0.001
Hypertension (yes/no)	40/48	38/42	0.791
Diabetes (yes/no)	41/47	44/36	0.276
Hyperlipidemia (yes/no)	43/45	41/39	0.757
Family history of CAD (yes/no)	23/65	17/63	0.712
Creatinine (μmol/l)	91.27 ± 10.95	95.92 ± 12.36	0.366
CRP (mg/L)	8.41 ± 2.95	12.41 ± 3.53	<0.001
D-dimer (mg/l)	0.48 ± 0.24	0.83 ± 0.24	<0.001
NT-proBNP (pg/ml)	306.13 ± 99.92	458.73 ± 94.39	<0.001
Door to balloon time (min)	118.26 ± 10.94	120.75 ± 11.51	0.726
**Culprit vessel (%)**			
Left anterior descending	37 (42.05%)	33 (41.25%)	
Left circumflex artery	12 (13.64%)	17 (21.25%)	
Right coronary artery	39 (44.31%)	29 (36.25%)	
Left main coronary artery	0 (0)	1 (1.25%)	
Number of implanted stents	1.09 ± 0.34	1.07 ± 0.31	0.742
**Medication after surgery (%)**			
Aspirin	88 (100.00%)	79 (98.75%)	0.784
Clopidogrel	85 (96.59%)	79 (98.75%)	0.751
ACEI or ARB	46 (52.27%)	47 (58.75%)	0.620
Statins	82 (93.18%)	75 (93.75%)	0.899
β-Blockers	36 (40.91%)	35 (43.75%)	0.717

Note: Data are expressed as n or mean ± standard deviation.

Abbreviations: BMI, body mass index; CRP, C-reactive protein.

### Serum expression levels of NT-proBNP and D-dimer

**Figure 1.**
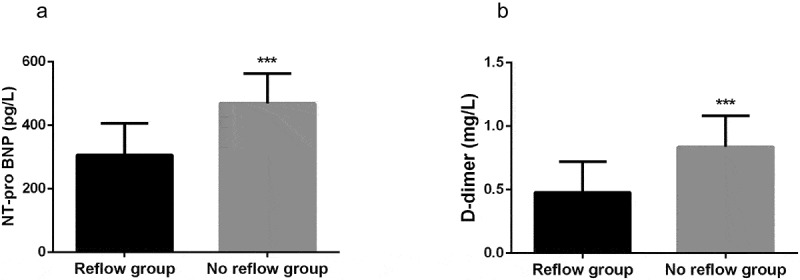
Expression level of serum NT-proBNP (a) and D-dimer (b) in ACS patients. NT-proBNP and D-dimer were significantly higher in no-reflow group than that in normal reflow group. ***P < 0.001

To investigate the expression of NT pro-BNP and D-dimer in the serum of patients, the expression levels of serum NT-proBNP and D-dimer in ACS patients after PCI were measured by Immunofluorescence double antibody sandwich method and enzyme-linked immunosorbent assay (ELISA). The results showed that the levels of NT-proBNP and D-dimer in the no-reflow patient group were significantly higher than those in the normal reflow patient group (Figure 1(a,b), *P* < 0.001).

### ROC curve analysis

**Figure 2.**
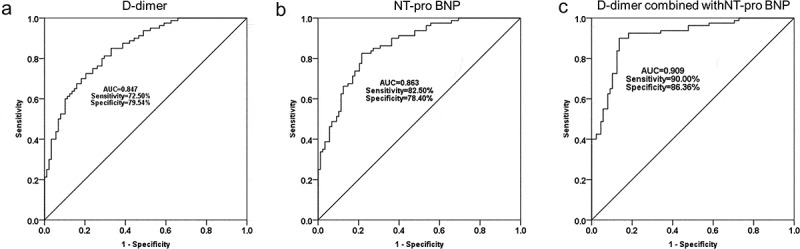
ROC curves were established to evaluate the predictive value of serum D-dimer (a), NT-proBNP (b) and D-dimer combined with NT-proBNP (c) for no reflow after PCI

In order to evaluate the predictive value of NT pro-BNP and D-dimer for non-reflow phenomenon after PCI, ROC curves were established to assess the predictive value of NT-proBNP and D-dimer for ACS patients without reflow after PCI. The level of serum D-dimer has predictive value for postoperative no reflow in ACS patients. The ROC curve had an AUC value of 0.847 with a sensitivity of 72.50% and a specificity of 79.54% (Figure 2a). In addition, the ROC curve of NT-proBNP had an AUC value of 0.863 with a sensitivity of 82.50% and a specificity of 78.40% (Figure 2b). It was worth noting that the AUC value of NT-proBNP combined with D-dimer significantly increased to 0.909, and the sensitivity and specificity also increased to 90.00% and 86.36%, respectively (Figure 2c).

### Logistic regression analysis

To explore the relationship between clinical indicators and no reflow after PCI, a logistic regression model was used to analyze the influencing factors of no-reflow in ACS patients after PCI. After the baseline data was incorporated in the model analysis, as shown in Table 2, it was found that preoperative D-dimer (OR = 3.433, 95%CI = 1.654–7.123, *P* = 0.001) and NT-proBNP (OR = 4.024, 95%CI = 1.944–8.329, *P* < 0.001) were independent influencing factors of no reflow after PCI.

**Table 2.** Association of different variables with the occurrence of reflow after acute coronary syndrome

Variables	OR	95% CI	*P* value
N (Male and female)	0.850	0.419–1.723	0.652
Age (years)	1.835	0.890–3.784	0.100
BMI (kg/m^2^)	0.551	0.274–1.107	0.094
Hypertension (n)	1.134	0.567–2.267	0.722
Diabetes (n)	1.357	0.680–2.708	0.386
Hyperlipidemia (n)	1.053	0.526–2.109	0.884
Smoker (n)	0.813	0.393–1.681	0.576
D-dimer (mg/l)	3.433	1.654–7.123	0.001
NT-proBNP (pg/ml)	4.024	1.944–8.329	<0.001

Abbreviation: BMI, body mass index.

### Levels of NT-proBNP and D-dimer in serum at different times

To evaluate the expression of NT-proBNP and D-dimer in serum of patients at different time, the levels of NT-proBNP and D-dimer in the patient’s serum were detected before PCI, immediately after surgery, 2 hours and 6 months after surgery. The results were shown in Tables 3 and Tables 4. The expression level of NT-proBNP in the no-reflow patient group was significantly enhanced compared with the normal reflow patient group before surgery, 2 hours after surgery and 6 months (*P* < 0.001). However, the NT-proBNP level of the no-reflow patient group increased when detected immediately after surgery, but the difference between groups was not very significant (*P* = 0.021), which might be due to the small sample size. For D-dimer, the level in the no-reflow patient group was significantly higher than that of the normal reflow patient group before surgery, immediately after surgery, 2 hours and 6 months after surgery (*P* < 0.001). In addition, it can be seen from the results that at 6 months after the surgery, the levels of NT-proBNP and D-dimer in the normal reflow and no-reflow patient group decreased compared with the preoperative level, indicating that the patients gradually recovered after PCI.

**Table 3.** Comparison of serum NT-proBNP levels at different times

Variables	All subjects (N = 168)		*P* value
	Reflow group (n = 88)	No-reflow group (n = 80)	
Preoperative	306.13 ± 99.92	458.73 ± 94.39	<0.001
Immediately after surgery	581.77 ± 114.07	626.97 ± 136.38	0.021
2 h after surgery	277.06 ± 22.29	634.84 ± 121.46	<0.001
6 months after surgery	227.05 ± 22.04	303.62 ± 24.53	<0.001

Note: Data are expressed as n or mean ± standard deviation.

**Table 4.** Comparison of serum D-dimer levels at different times

Variables	All subjects (N = 168)		*P* value
	Reflow group (n = 88)	No-reflow group (n = 80)	
Preoperative	0.48 ± 0.24	0.84 ± 0.24	<0.001
Immediately after surgery	1.31 ± 0.26	2.10 ± 0.59	<0.001
2 h after surgery	0.42 ± 0.04	2.36 ± 0.61	<0.001
6 months after surgery	0.38 ± 0.02	0.50 ± 0.10	<0.001

Note: Data are expressed as n or mean ± standard deviation.

## Discussion

No reflow refers to the phenomenon of myocardial reperfusion insufficiency despite the removal of mechanical obstruction after PCI and the recanalization of coronary angiography. The mechanism of no-reflow after PCI is not completely clear at present, and some studies have proposed the following mechanism: 1) vascular endothelial dysfunction; 2) microvascular dysfunction; 3) thromboembolism; 4) ischemic injury; 5) reperfusion injury, etc. [18–20]. No-reflow is one of the dangerous complications after emergency PCI and is directly related to the size of myocardial infarction, with an incidence rate of about 25% [21]. Currently, there are various drugs and devices to deal with the possibility of no-reflow, but an early accurate prediction of no-reflow may reduce the incidence of no reflow [22]. In the present study, the levels of NT-proBNP and D-dimer in no-reflow patients after PCI were significantly higher than those in normal reflow patients. The ROC curve showed that the AUC value of NT-proBNP combined with D-dimer increased to 0.999, and the Logistics regression analysis suggested that NT-proBNP and D-dimer were independent predictors of no-reflow after PCI.

Serum concentrations of atrial natriuretic peptide (ANP), brain natriuretic peptide (BNP), NT-proANP, and NT-proBNP have been reported to be associated with long-term survival after acute myocardial infarction. It was reported that no-reflow phenomenon in STEMI patients could be predicted according to the high level of serum BNP on admission [23]. Previous studies have shown that the increased expression of NT-proBNP in the blood of ACS patients was a strong predictor of adverse cardiovascular events. For example, Olmand et al. demonstrated that the blood levels of NT-proBNP were significantly associated with early death in patients with acute myocardial infarction [24]. Heeschen et al. reported that the serum level of NT-proBNP was elevated in ACS patients, and continuous measurement of NT-proBNP level in ACS patients could effectively predict the disease progression of patients [25]. It was reported by Hong et al. that the serum NT-proBNP level increased significantly in patients with ST-Segment elevation acute myocardial infarction (STEMI) without reflow after PCI and speculated that NT-proBNP might be a strong predictor of no-reflow after PCI in STEMI patients [12]. Our results are consistent with those of Hong et al., suggesting that preoperative high level of serum NT-proBNP was an independent predictor of no-reflow in ACS patients after PCI.

The unstable plaque breaks in the coronary artery and forms a thrombus, which eventually leads to the development of ACS [26]. D-dimer is a degradation product of fibrin found in the blood and is associated with thromboembolism [27]. The increase in the level of serum D-dimer directly reflected the body hypercoagulable state and thrombus load state, thereby indirectly reflecting the size of thrombus formation [28]. Several studies have shown that D-dimer may have good diagnostic and prognostic values for AMI and unstable angina pectoris. High levels of D-dimer have been associated with poor outcomes in ACS, and studies by Akgul et al. have demonstrated that high level of preoperative serum D-dimer is positively associated with increased mortality in STEMI patients after PCI [14]. In this study, preoperative D-dimer level of patients with no reflow was higher than that of patients with normal reflow, and ROC curve results confirmed that D-dimer had a certain significance in predicting the occurrence of no reflow. Similarly, this result was supported by the research of Erkol et al., it was reported that serum D-dimer level of no-reflow patients was higher than that of normal reflow patients, which indicated that the D-dimer level at admission could independently predict the occurrence of no reflow after PCI. In the research of Gao et al., D-dimer and Endothelin-1 (ET-1) have the value of independently predicting the non-reflow of ACS patients with type II diabetes after PCI, and their predictive significance was improved when D-dimer and ET-1 were combined [29]. NT-proBNP and D-dimer, two factors with independent predictive value, were selected for the first time to investigate the predictive value of these two factors for no-reflow after PCI in our study. Based on the results of the ROC curve, it can be seen that the AUC values of NT-proBNP and D-dimer were 0.863 and 0.847, respectively. However, the AUC value of the curve increased to 0.909 when NT-proBNP was combined with D-dimer. The above results indicate that NT-proBNP combined with D-dimer improves the predictive value of no-reflow after PCI.

## Conclusion

In summary, preoperative NT-proBNP combined with D-dimer had a certain predictive value in predicting the occurrence of no reflow after PCI. This is of great significance to guide diagnosis and medication. Similarly, this study may provide some experimental basis for other studies on no-reflow phenomenon in the future. The deficiency of this study is that the sample size of subjects is too small, and the sample needs to be further expanded. Larger clinical trials are still needed to verify the predictive ability of the combination of multiple factors.

## Supplementary Material

Supplemental Material

## Data Availability

The data that support the findings of this study are available from the corresponding author upon reasonable request.

## Highlights

1. The levels of NT pro-BNP and D-Dimer in serum of non-reflow patients after PCI were both elevated.

2. D-dimer combined with NT pro-BNP showed high diagnostic value in non-reflow patients after PCI.

3. NT-proBNP and D-dimer were independent predictors of the occurrence of no reflow in the total population.
