# Supplementary figures and images for: Predictive value of N-terminal pro-B-type natriuretic peptide (NT-pro BNP) combined with D-dimer for no-reflow phenomenon in patients with acute coronary syndrome after emergency of percutaneous coronary intervention
Source: Bioengineered. 2021 Oct 26;12(1):8614–21. doi: 10.1080/21655979.2021.1988361 (PMC8806976; doi:10.1080/21655979.2021.1988361)

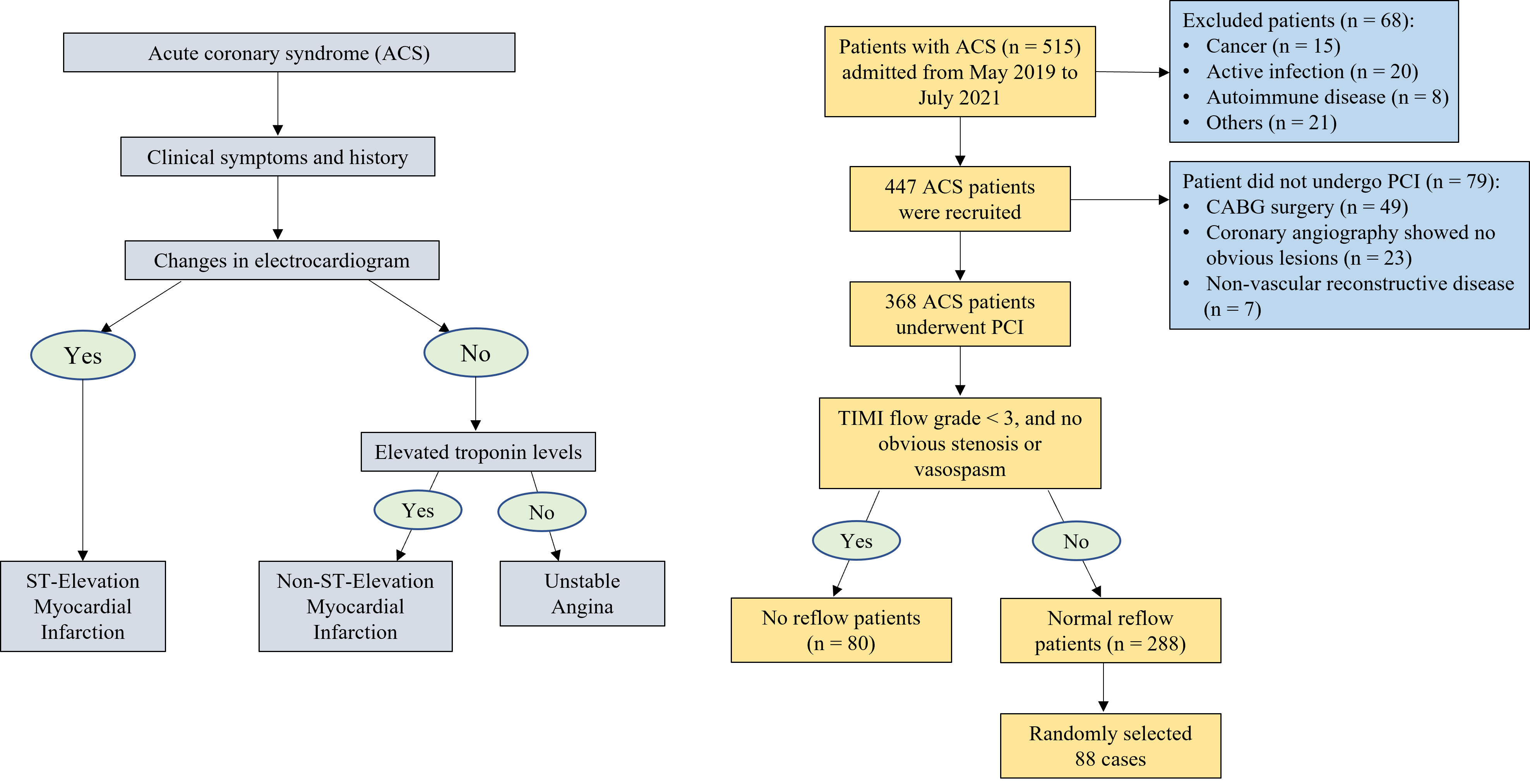

Supplement: Supplemental Material [file KBIE_A_1988361_SM3673.tif]
